# Online Patient Attitudes Toward Cutaneous Immune-Related Adverse Events Attributed to Nivolumab and Pembrolizumab: Sentiment Analysis

**DOI:** 10.2196/53792

**Published:** 2024-05-02

**Authors:** Camille M Powers, Andrew K Yang, Hannah Verma, Jeremy Orloff, Austin J Piontkowski, Nicholas Gulati

**Affiliations:** 1 Department of Dermatology Icahn School of Medicine at Mount Sinai New York, NY United States

**Keywords:** immune checkpoint inhibitor, immunotherapy, pembrolizumab, nivolumab, cutaneous immune-related adverse event, medical dermatology, oncology, sentiment analysis

## Introduction

Online forums provide patients with platforms to connect, share experiences, and learn about conditions and treatments [1]. Drugs.com, one publicly available website, hosts patient reviews on medication-related adverse events. Since 2011, immune checkpoint inhibitors (ICIs) have revolutionized cancer therapy. Increasing use of ICIs has led to more patients experiencing ICI-induced cutaneous immune-related adverse events (cirAEs), occurring in 30%-60% of patients [2]. However, many nondermatologist physicians feel unequipped to manage cirAEs, making dermatologists with expertise in skin-related conditions crucial in detection, diagnosis, and management [3]. Furthermore, despite cirAEs being linked to improved survival and treatment response, they are frequently distressing to patients. Patient reviews of ICIs could offer insights into attitudes and expectations about cirAEs during cancer treatment, aiding physician-patient education. Previous literature highlights the potential for natural language processing to provide valuable insights [4,5]. Our study aims to characterize public online oncology patients’ attitudes toward dermatologic symptoms (DSs) during ICI treatment and explore whether patients mentioning DSs also report improved cancer outcomes.

## Methods

### Overview

Data on ICIs nivolumab and pembrolizumab were collected from Drugs.com using Python (Multimedia Appendix 1). Reviews were screened for DSs using the following terms: skin, dermatitis, rash, blisters, dry, itch, and peeling. Sentiment scores were derived using cardiffnlp/twitter-roberta-base-sentiment-latest, a Robustly Optimized Bidirectional Encoder Representations From Transformers (RoBERTa)–based artificial intelligence technique that captures contextual semantics [6,7]. Two-tailed Mann-Whitney *U* tests compared median ratings and sentiment scores in DS-containing reviews versus those without. Positive cancer outcomes were determined by manual review, including the words remission, gone, resolution, shrunk/shrink, smaller, reduction, disappeared, cancer-free, and saved. Significance was evaluated using a Fisher test. After examining the distribution (Table 1), patient reviews were divided into three score ranges (1-3, 4-7, and 8-10 out of 10) by dividing the maximum rating of 10 into thirds and rounding to the nearest nonoverlapping whole number. A row-wise Fisher test was used to compare DS-containing versus non–DS-containing reviews across the three score groups, with a Benjamini-Hochberg procedure to adjust *P* values.

**Table 1:** Distribution of patient ratings of nivolumab and pembrolizumab.

	Scores 1-3, n	Scores 4-7, n	Scores 8-10, n	Total reviews, n
Nivolumab	52	6	43	101
Pembrolizumab	121	14	56	191
All	173	20	99	292

### Ethical Considerations

Given the publicly available nature of the data, no institutional review board approval was warranted for this study. We prioritized patient privacy and minimized potential harms by anonymizing data, analyzing all reviews, transparently documenting our methods, and comparing findings to existing literature.

## Results

**Figure 1:**
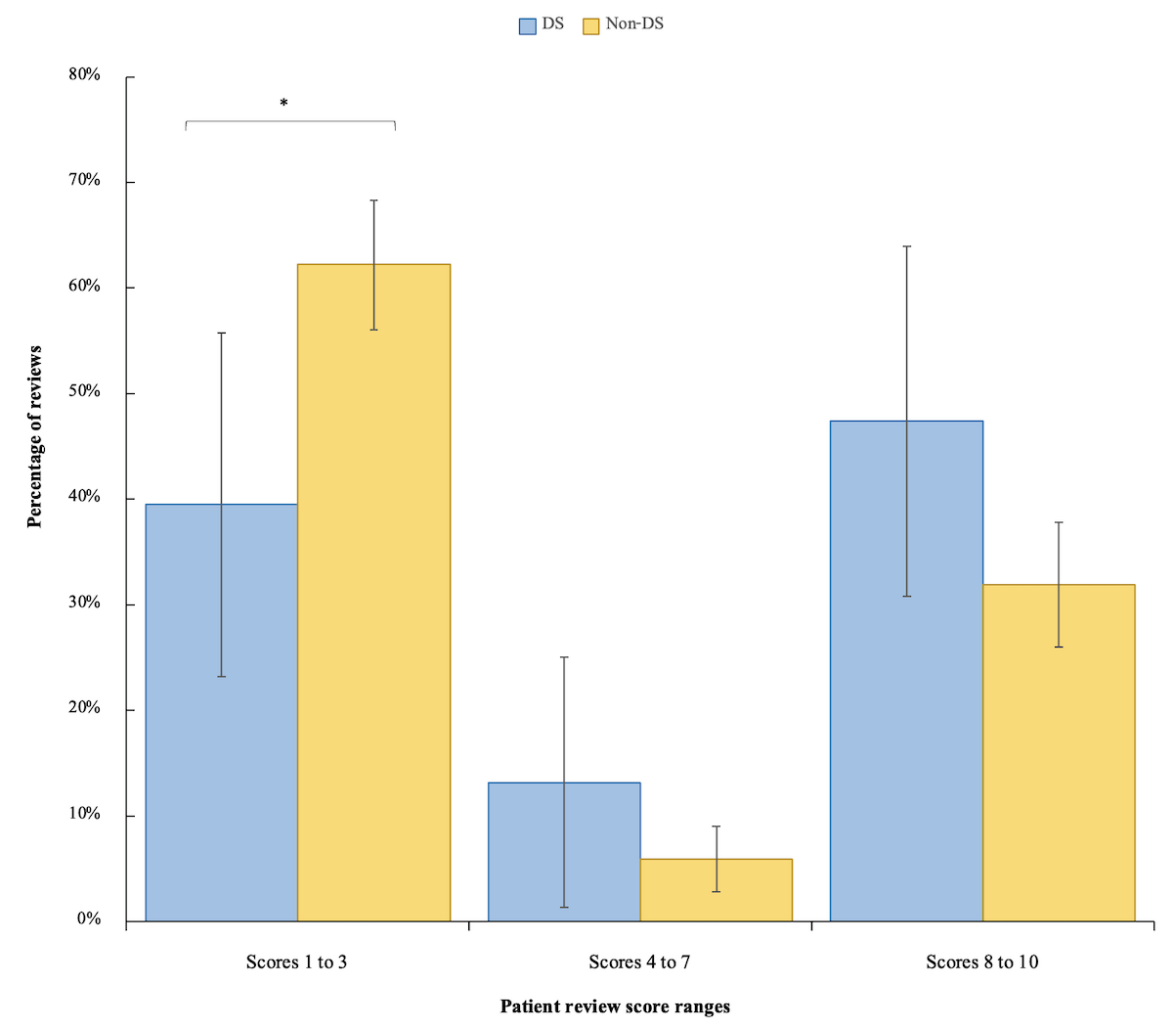
Percentages of patient reviews mentioning DSs versus not mentioning DSs in each score range. All scores are out of 10 and were extracted from patient ratings of nivolumab or pembrolizumab on Drugs.com. The error bars represent 95% CIs, calculated by the Clopper-Pearson method. DS: dermatologic symptom. *Significant by row-wise Fisher test, defined as *P*<.05.

Of 292 reviews, 38 mentioned DSs (21 for nivolumab and 17 for pembrolizumab), while 254 did not. The distribution of ratings was heavily skewed toward extremes, but a handful of reviews were moderately rated. The top two ICI indications were non–small cell lung cancer (117/292, 60.9%) and melanoma (52/292, 17.8%). Mean patient ratings were significantly higher for nivolumab than pembrolizumab (mean 4.97, SD 4.08 vs mean 3.85, SD 3.79 out of 10; *P*=.02). DS-containing reviews had significantly higher patient ratings (median 6.5, IQR 1-10 vs median 1.0, IQR 1-9 out of 10; *P*=.007). A trend toward higher sentiment scores was exhibited in DS-containing reviews, though it did not reach statistical significance (*P*=.07). Overall, 16 of 38 (42%) DS-containing reviews compared to 40 of 254 (15.8%) non–DS-containing reviews self-reported positive cancer outcomes, including remission or tumor size reduction (*P*<.001). A significantly lower proportion of DS-containing compared to non–DS-containing reviews had ratings of 1 to 3 out of 10 (15/38, 39% vs 158/254, 62.2%; *P*=.04). Higher proportions of DS-containing reviews were in the score ranges of 4-7 and 8-10, but these did not reach statistical significance (*P*=.16 and *P*=.10, respectively; Figure 1).

## Discussion

In summary, DS-containing reviews correlated with higher patient ratings and more self-reported positive cancer outcomes. Nivolumab was rated higher than pembrolizumab. The FDA reports the prevalence of pembrolizumab- and nivolumab-associated DS as 13.57% and 12.61%, respectively, aligning with the 13% (38/292) of DS-mentioning reviews in our study [8]. While not compared directly, pembrolizumab and nivolumab have both been associated with improved patient-reported quality of life [9,10]. CirAE development was associated with more self-reported positive cancer outcomes, reinforcing the presence of DS as a promising indicator of treatment efficacy [2]. Higher patient ratings were likely influenced by improvements in cancer. Thus, patient counseling by dermatologists regarding the prognostic value of cirAEs may improve patient satisfaction.

Online patient reviews have limitations. They skew toward younger English-speaking individuals with higher digital literacy and extremely positive or negative experiences. Patient reviews include subjective accounts of cancer diagnoses and improvement, lacking medical history and social context, may be emotionally biased or inaccurate and represent only a snapshot in time. Sentiment analysis tools may also carry biases; for example, our study’s chosen model was trained on social media data, not health care data [7]. However, we believe this model is applicable due to the online and short-form nature of the reviews. Analyzing patient reviews offers direct feedback to clinicians and informs unmet patient needs. Future research could involve prospective data collection to quantify patients’ subjective experiences alongside objective clinical cirAE grading to better guide the treatment of oncodermatologic conditions.

## Appendix

Multimedia Appendix 1 Supplemental methods.

## Abbreviations

cirAE: cutaneous immune-related adverse event
DS: dermatologic symptom
ICI: immune checkpoint inhibitor
RoBERTa: Robustly Optimized Bidirectional Encoder Representations From Transformers

## Data Availability

The data underlying this article will be shared upon reasonable request to the corresponding author.
