# Supplementary material for: Online Patient Attitudes Toward Cutaneous Immune-Related Adverse Events Attributed to Nivolumab and Pembrolizumab: Sentiment Analysis
Source: JMIR Dermatol. 2024 May 2;7:e53792. doi: 10.2196/53792 (PMC11099803; doi:10.2196/53792)
Supplement: Multimedia Appendix 1 [file derma_v7i1e53792_app1.docx]

Supplemental Methods

Data Sourcing and Cleaning

Reviews were scraped from drugs.com using a Selenium web scraper written in Python 3. The scraper captured drug, reviewer screen name, date of review, review text, rating (of the drug), and thumbs up count (as an indicator of review helpfulness). Reviews without ratings or incomplete review text were removed.

Sentiment Analysis

Valence Aware Dictionary and sEntiment Reasoner (VADER) and RoBERTa were tested for sentiment analysis. The ‘cardiffnlp/twitter-roberta-base-sentiment-latest’ fine-tuned implementation of RoBERTa was utilized for sentiment analysis due to its superior R^2 value, with respect to given patient rating. Additionally, transformer based models are likely more capable of analyzing the complex sentiment towards cancer medications. An example of such nuance can be found within the following review:

“Been on Nivolumba [sic] since July 31, every 2 weeks. As of Sept.14 I've lost about10 lbs, have loss of appetite, cough, and fatigue. I will be starting acupuncture today (9/15) and believe this will help me. I went through 6 weeks (every day)of radiation and chemo last year and started acupuncture at the very beginning of treatments and never had any side effects except loss of hair and I walked 2 miles every day while under going treatments. I'm very encouraged and believing that Nivolumba [sic] will eliminate my cancer. I can't compare the side effects of my treatments last year with Nivolumba [sic] because of the timely maner of acupuncture."

VADER: -0.78 (-1 to 1)

RoBERTa: 0.36 (-1 to 1)

Indicated Rating: 9/10

A scaled average was extracted from the model output. Large language models may be over-tuned to specific purposes and may exhibit their own biases based on training data. We acknowledge this as a limitation of the chosen model. However, due to the character limit of drugs.com and internet format, we believe that the ‘cardiffnlp/twitter-roberta-base-sentiment-latest' model is appropriately generalizable. In addition, finetuning a model has its own resource and data limitations.

A manual review was also conducted to analyze positive cancer response and to evaluate the performance of the large language model. We also analyzed the sentiments and content of reviews and made sure no identifying information was included in our reporting of data to uphold patients’ autonomy, privacy, and dignity.
